# Impact of acute kidney injury on long-term adverse outcomes in obstructive uropathy

**DOI:** 10.1038/s41598-021-03033-0

**Published:** 2021-12-08

**Authors:** Jihyun Yang, Bong Gyun Sun, Hyeon-Jin Min, Young-Bin Son, Tae Bum Kim, Jonghyun Lee, Se Won Oh, Myung-Gyu Kim, Won Yong Cho, Shin Young Ahn, Gang-Jee Ko, Young Joo Kwon, Jin Joo Cha, Young Sun Kang, Dae Ryong Cha, Sang-Kyung Jo

**Affiliations:** 1 Department of Internal Medicine, Korea University Anam Hospital, Korea University Medical College, Koreadae-Ro 73, Sungbuk-Gu, Seoul, Korea; 2 Department of Internal Medicine, Korea University Guro Hospital, Gurodong-Ro 148, Guro-Gu, Seoul, Korea; 3 Department of Internal Medicine, Korea University Ansan Hospital, Jeokgeum-Ro 123, Danwon-Gu, Ansan, Korea

**Keywords:** Nephrology, Risk factors, Urology

## Abstract

Obstructive uropathy is known to be associated with acute kidney injury (AKI). This study aimed to investigate the etiologies, clinical characteristics, consequences and also assess the impact of AKI on long-term outcomes. This multicenter, retrospective study of 1683 patients with obstructive uropathy who underwent percutaneous nephrostomy (PCN) analyzed clinical characteristics, outcomes including progression to end-stage kidney disease (ESKD), overall mortality, and the impact of AKI on long-term outcomes. Obstructive uropathy in adults was most commonly caused by malignancy, urolithiasis, and other causes. AKI was present in 78% of the patients and was independently associated with preexisting chronic kidney disease (CKD). Short-term recovery was achieved in 56.78% after the relief of obstruction. ESKD progression rate was 4.4% in urolithiasis and 6.8% in other causes and older age, preexisting CKD, and stage 3 AKI were independent factors of progression. The mortality rate (34%) was highly attributed to malignant obstruction (52%) stage 3 AKI was also an independent predictor of mortality in non-malignant obstruction. AKI is a frequent complication of adult obstructive uropathy. AKI negatively affects long-term kidney outcomes and survival in non-malignant obstructions. A better understanding of the epidemiology and prognostic factors is needed for adult obstructive uropathy.

## Introduction

Obstructive uropathy is a common clinical condition that leads to acute or chronic kidney failure. Obstruction to urine flow can occur in any location in the urinary tract, and may be acute or chronic, unilateral or bilateral, and partial or complete. While obstructive uropathy is known to be a frequent cause of CKD/ESKD in children, it is known to be associated with AKI in adults^1,2^.

Obstruction to urine flow increases the pressure proximal to the obstruction, consequently decreasing the intraglomerular pressure gradient, which leads to a decreased glomerular filtration rate (GFR)^3^. Activation of the renin–angiotensin–aldosterone system mediated by renal vasoconstriction further reduces the renal blood flow and GFR^4^. In addition to the hemodynamic effects, abnormalities in the cellular paradigm, including the development of interstitial inflammation, tubular atrophy, and fibrosis, also contribute to kidney injury^5^.

The incidence, etiologies, and clinical manifestations of obstructive uropathy are likely to vary according to the patients’ age, sex, or underlying disease conditions. For example, urolithiasis is known to be a common cause in young adults, whereas benign prostatic hypertrophy and retroperitoneal or pelvic malignancies are more common in the elderly^6^. Although early recognition and prompt relief of obstruction can reverse the injury and restore function completely, delayed diagnosis due to the absence of specific symptoms, superimposed infection, or underlying comorbidities might affect the short- and long-term kidney outcomes.

In contrast to pediatric obstructive uropathy, which is mainly caused by congenital abnormalities of the urinary tract, there have been no extensive epidemiological data regarding the clinical characteristics and long-term outcomes of obstructive uropathy in adults. In this study, we conducted a multicenter retrospective analysis of 1683 consecutive patients who underwent PCN for obstructive uropathy in Korea. This study aimed to determine the etiologies, clinical manifestations, outcomes, and impact of superimposed AKI on these outcomes.

## Results

### Study subjects

**Figure 1:**
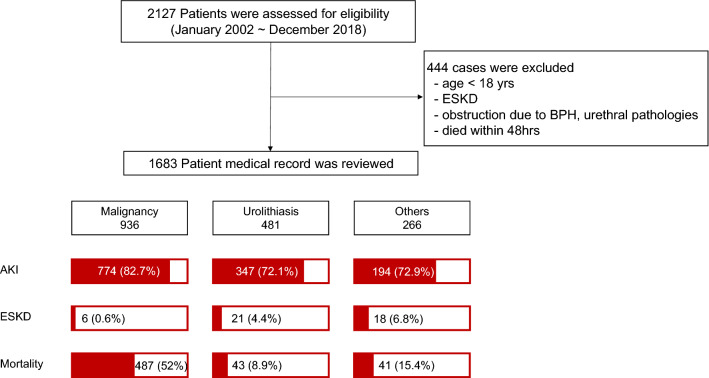
Enrollment and outcomes. Among 2127 patients who underwent PCN for obstructive uropathy between January 1, 2002 and December 31, 2018, in three tertiary hospitals were assessed for the inclusions, patients were excluded and finally 1683 patients were enrolled for assessment. According to PCN divided into three etiologies, the short-term renal outcome, long term renal outcome and all-cause mortality were schematized.

Among the 2127 patients who underwent PCN, 444 who were under 18 years, previously diagnosed with ESKD, died within 48 h of admission or with lower urinary tract obstruction were excluded and 1683 patients were finally analyzed. Multiple episodes of PCN insertion were counted as one. A total of 1683 patients were analyzed (Fig. 1).

### Etiologies of obstructive uropathy in adults

The patients were divided into three groups according to the etiology of obstruction. The most common cause was malignancy (n = 936, 55.6%), followed by urolithiasis (n = 481, 28.6%), and other causes (n = 266, 15.8%) (Table 1). Other causes included ureteral stricture, intra-abdominal abscess, retroperitoneal fibrosis, vesicoureteral reflux, and renal tuberculosis. Among the malignancies, metastatic colorectal cancer (n = 245, 26.2%) was the most common, followed by gastric cancer (n = 188, 20.1%), bladder cancer (n = 143, 15.3%), uterine cervical cancer (n = 97, 10.4%), and prostate cancer (n = 68, 7.3%) (Supplementary Table S1).

**Table 1:** Demographic and clinical characteristics of the study cohort according to etiologies.

	Malignancy (N = 936, 55.6%)	Urolithiasis (N = 481, 28.6%)	Others (N = 266, 15.8%)	p-value	
Gender (male)	496 (53%)	236 (49.1%)	92 (34.6%)^§†^	< 0.001	
Age [years]	62.99 [24, 95]	62.37 [23, 95]	59.06 [21, 93]^§†^	< 0.001	
**Comorbidities**					
DM	224 (23.9%)	210 (43.7%)*	98 (36.8%)^§^	< 0.001	
HTN	376 (40.2%)	296 (61.5%)*	125 (47%)^†^	< 0.001	
Chronic liver disease	79 (8.4%)	53 (11.0%)	21 (7.9%)	0.204	
IHD	39 (4.2%)	41 (8.5%)*	16 (6.0%)	0.003	
PAOD	39 (4.2%)	28 (5.8%)	14 (5.3%)	0.320	
Heart failure	25 (2.7%)	19 (4.0%)	14 (5.3%)	0.098	
Dementia	13 (1.4%)	22 (4.6%)*	6 (2.3%)	0.001	
Hemiparesia	6 (0.6%)	19 (4.0%)*	3 (1.1%)^†^	< 0.001	
COPD	59 (6.3%)	31 (6.4%)	11 (4.1%)	0.350	
CKD	204 (21.8%)	110 (22.9%)	86 (32.3%)^§†^	0.002	
**Laboratory test**					
Hb [g/dL]	10.02 [3.40, 16.10]	11.7 [4.40, 19.10]*	10.74 [4.50, 16.80]^§†^	< 0.001	
WBC [× 10^3^/µL]	8.74 [0.35, 45.50]	12.56 [0.89, 56.00]*	10.23 [1.56, 35.55]^§†^	< 0.001	
Platelet [× 10^3^/µL]	239 [7.4, 844.0]	205 [13.0, 649.0]*	223.5 [17.0, 609.0]^†^	< 0.001	
Na [mmol/L]	136.4 ± 4.99	137.06 ± 4.82	136.6 ± 5.67	0.176	
K [mmol/L]	4.48 [2.02, 9.74]	4.27 [2.60, 8.39]*	4.40 [2.67, 7.38]	< 0.001	
Cl [mmol/L]	101.86 [68.0, 123.0]	103.40 [73.0, 134.0]*	103.17 [75.0, 117.4]^§^	< 0.001	
Total CO_2_ [mmol/L]	22.4 ± 5.05	20.98 ± 5.02*	20.5 ± 5.3^§^	< 0.001	
Baseline Cr [mg/dL]	0.87 ± 0.55	0.93 ± 0.64	1.06 ± 0.69^§^	0.001	
Baseline eGFR	88.25 [1.59, 188.96]	84.08 [7.99, 184.42]	79.10 [10.98, 144.70]^§^	0.002	
CRP [mg/L]	66.04 [0.11, 444.57]	108.41 [0.13, 435.50]*	81.82 [0.10, 348.68]^§†^	< 0.001	
Procalcitonin[ng/mL]	6.03 [0.02, 100.00]	26.68 [0.04, 200.00]*	24.65 [0.03, 100.00]^§^	< 0.001	
Glucose [mg/dL]	127.4 [31.0, 581.0]	150.8 [30.0, 743.0]*	147.1 [62.0, 824.0]^§^	< 0.001	
Protein [g/dL]	6.59 ± 0.86	6.58 ± 0.92	6.67 ± 0.95	0.326	
Albumin [g/dL]	3.52 [1.50, 6.00]	3.58 [1.10, 5.50]	3.52 [1.80, 4.90]	0.175	
Uric acid [mg/dL]	6.3 ± 3.3	6.3 ± 3.1	6.4 ± 2.9	0.994	
**AKI**	774 (82.7%)	347 (72.1%)*	194 (72.9%)^§^	< 0.001	
Stage 1	283 (36.6%)	131 (37.8%)	76 (39.2%)		
Stage 2	176 (22.7%)	103 (29.7%)	41 (21.1%)		
Stage 3	315 (40.7%)	113 (32.6%)	77 (39.7%)		

*DM* diabetes mellitus, *HTN* hypertension, *IHD* ischemic heart disease, *PAOD* peripheral arterial occlusive disease, *COPD* chronic obstructive pulmonary disease, *CKD* chronic kidney disease, *Hb* hemoglobin, *eGFR* estimated glomerular filtration rate, *CRP* C-reactive protein, *AKI* acute kidney injury.

*Malignancy vs. urolithiasis, ^§^malignancy vs. others, ^†^urolithiasis vs. others, p-value < 0.05.

Patients with malignancy-associated obstruction were more likely to be older, male, have lower hemoglobin levels, and have AKI. In contrast, patients with urolithiasis had a higher prevalence of diabetes, hypertension, ischemic heart disease, hemiparesis, and dementia. The prevalence of underlying CKD was the highest in the other causes group, with a significantly lower baseline eGFR. Inflammatory markers, including levels of C-reactive protein (CRP), procalcitonin, and leukocyte counts, were also significantly higher in patients with urolithiasis or other causes, suggesting that these patients might have superimposed urinary tract infection (Table 1).

### Obstructive uropathy associated with AKI

Among the 1683 patients who underwent PCN, 1313 (78.0%) initially presented with AKI. A total of 216 patients (16.4%) required temporary dialysis. Patients with AKI were more likely to be older, male, and have preexisting CKD with a significantly lower baseline eGFR, and lower hemoglobin, albumin, and total CO_2_ levels. However, patients with AKI had a significantly higher leukocyte count, CRP, procalcitonin, and uric acid levels than those without. Furthermore, the percentage of malignancy as the etiology of obstruction was significantly higher in patients with AKI (Table 2).

**Table 2:** Demographic and clinical characteristics of the study cohort according to the presence of AKI.

	No AKI (n = 368, 21.9%)	AKI (n = 1315, 78.1%)	p-value
Gender (Male)	159 (43.2%)	665 (50.6%)	0.013
Age (year)	58.90 ± 14.82	63.17 ± 14.10	< 0.001
**Comorbidities**			
DM	110 (30.2%)	421 (32.0%)	0.526
HTN	158 (42.9%)	639 (48.6%)	0.059
Chronic liver disease	35 (9.5%)	118 (9.0%)	0.759
IHD	16 (4.3%)	80 (6.1%)	0.252
PAOD	17 (4.6%)	64 (4.9%)	0.486
HF	9 (2.4%)	49 (3.7%)	0.262
Dementia	4 (1.1%)	37 (2.8%)	0.057
Hemiparesia	6 (1.6%)	22 (1.7%)	0.585
COPD	16 (4.3%)	85 (6.5%)	0.138
Connective tissue disease	15 (4.1%)	49 (3.7%)	0.758
CKD	37 (10.1%)	363 (27.6%)	< 0.001
**Laboratory test**			
Hb [g/dL]	11.4 ± 2.26	10.3 ± 2.23	< 0.001
WBC [× 10^3^/µL]	8.55 [0.35, 32.03]	10.39 [0.46, 46.70]	< 0.001
Platelet [× 10^3^/µL]	249.1 [9.5, 837.0]	241.3 [7.4, 844.0]	0.117
Na [mmol/L]	138.4 [114.0, 147.0]	136.2 [107.2, 158.0]	< 0.001
K [mmol/L]	4.09 [2.02, 6.20]	4.49 [2.50, 9.74]	< 0.001
Cl [mmol/L]	103.1 [81.0, 115.0]	102.3 [68.0, 134.0]	0.041
Total CO_2_ [mmol/L]	24.5 [10.3, 48.1]	21.3 [4.1, 41.4]	< 0.001
Baseline Cr [mg/dL]	0.8 [0.17, 5.93]	1.0 [0.10, 6.40]	< 0.001
Baseline eGFR	94.73 [9.40, 157.14]	83.80 [7.99, 188.96]	< 0.001
CRP [mg/L]	57.25 [0.23, 383.39]	87.39 [0.10, 444.57]	< 0.001
Procalcitonin [ng/mL]	7.64 [0.03, 87.90]	20.64 [0.02, 200.0]	< 0.001
Glucose [mg/dL]	133.9 ± 64.5	138.4 ± 72.5	0.379
HbA1c [% of THb]	6.96 ± 1.75	6.57 ± 1.86	0.064
Protein [g/dL]	6.79 [3.30, 9.20]	6.56 [3.20, 9.70]	< 0.001
Albumin [g/dL]	3.72 ± 0.59	3.49 ± 0.60	< 0.001
Uric acid [mg/dL]	4.8 [1.3, 14.8]	6.7 [1.1, 29.1]	< 0.001
**Etiology of obstruction**			< 0.001
Malignancy	162 (44.0%)	774 (58.9%)	
Stone	134 (36.4%)	347 (26.4%)	
Others	72 (19.3%)	194 (14.8%)	

*DM* diabetes mellitus, *HTN* hypertension, *IHD* ischemic heart disease, *PAOD* peripheral arterial occlusive disease, *COPD* chronic obstructive pulmonary disease, *CKD* chronic kidney disease, *Hb* hemoglobin, *eGFR* estimated glomerular filtration rate, *CRP* C-reactive protein, *AKI* acute kidney injury.

Using the decision tree model, we identified some relevant risk factors for AKI and performed a logistic regression analysis. Preexisting CKD, presence of hyponatremia (serum Na ≤ 135 mEq/L), and higher uric acid level at initial presentation were found to be independent risk factors for AKI in patients with obstructive uropathy (Table 3).

**Table 3:** Logistic regression analysis of factors associated with AKI.

	Univariate			Multivariate		
	HR	95% CI	p-value	HR	95% CI	p-value
Age	1.02	1.01–1.03	< 0.001			
Sex	0.76	0.60–0.96	0.018			
HTN	1.22	0.97–1.54	0.093			
DM	1.08	0.84–1.39	0.567			
CKD	3.37	2.34–4.85	< 0.001	2.03	1.01–4.0	0.04
IHD	1.43	0.82–2.47	0.21			
Chronic liver disease	1.07	0.72–1.59	0.75			
Hb	0.82	0.77–0.86	< 0.001			
CRP	1.04	1.01–1.07	0.02	1.68	0.96–2.95	0.07
Hyponatremia (serum Na ≤ 135 mEq/L)	2.97	2.1–4.12	< 0.001	2.28	1.23–4.21	0.01
Albumin	0.52	0.41–0.65	< 0.001			
Hyperuricemia (serum uric acid ≥ 6.0 mg/dL)	1.32	1.23–1.42	< 0.001	2.15	1.21–3.84	0.01

Multiple logistic regression analysis was conducted after adjustment for sex, age, hypertension, diabetes mellitus, ischemic heart disease, chronic liver disease, chronic kidney disease (eGFR ≤ 60 ml/min/1.73 m^2^), Hb, hyponatremia (serum Na ≤ 135 mg/d), CRP ≥ 6 mg/dL, albumin, and hyperuricemia (serum uric acid ≥ 6.0 mg/dL).

*HTN* hypertension, *DM* diabetes mellitus, *CKD* chronic kidney disease, *IHD* ischemic heart disease, *Hb* hemoglobin, *CRP* C-reactive protein.

### Kidney outcomes of obstructive uropathy

Of the 1313 patients who developed AKI, 746 (56.8%) showed short-term functional recovery within 7 days after PCN, and there was no difference in the recovery rates across the different etiologies of obstruction (Table 4). Logistic regression analysis showed that only the presence of preexisting CKD was an independent factor for the failure of short-term recovery (Supplementary Table S2).

**Table 4:** Kidney outcomes of obstructive uropathy.

	Malignancy (N = 936)			Stone (N = 481)			Others (N = 266)			p-value
AKI	774 (82.3%)			347 (72.1%)			194 (72.9%)			0.001
AKI Recovery	419 (54.1%)			223 (64.3%)			104 (53.6%)			0.84
Progression to ESKD	6 (0.6%)			21 (4.4%)			18 (6.8%)			0.02
	No AKI (N = 162)	AKI (N = 774)	p-value	No AKI (N = 134)	AKI (N = 347)	p-value	No AKI (N = 72)	AKI (N = 194)	p-value	
	1 (0.6%)	5 (0.7%)	0.91	0 (0%)	21 (6.1%)	0.003	2 (2.8%)	16 (8.2%)	0.01	

*AKI* acute kidney injury, *ESKD* end-stage kidney disease.

Regarding long-term kidney outcomes, 45 patients (2.67%) progressed to ESKD. The progression rates in patients with urolithiasis and other causes were 4.4% and 6.8%, respectively, while that of malignant obstruction was only 0.6% (Table 4). This may be related to a higher cancer-related death rate. Due to huge differences, we separately analyzed the risk factors of ESKD progression between malignant and non-malignant causes of obstruction.

Logistic regression analysis revealed that older age, preexisting CKD, and stage 3 AKI were independent factors leading to the progression to ESKD. The presence of CKD and stage 3 AKI showed a 19-fold and fivefold increased risk of ESKD, respectively (Table 5).

**Table 5:** Logistic regression analysis of factors associated with ESKD progression (non-malignant obstruction).

	Univariate			Multivariate		
	OR	95% CI	p-value	OR	95% CI	p-value
Age	4.61	1.1–19.32	0.03	5.05	2.24–11.35	< 0.001
Sex	1.11	0.81–1.51	0.52	0.77	0.37–1.6	0.48
HTN	1.03	0.75–1.40	0.86	1.52	0.64–3.5	0.32
DM	2.68	1.37–5.25	0.004	1.19	0.97–4.09	0.11
CKD	15.16	6.57–34.99	< 0.001	19.17	7.5–48.99	< 0.001
AKI stage 3	9.17	2.05–40.93	0.01	5.31	1.05–26.78	0.04

Multiple logistic regression analysis was conducted after adjustment for sex, age over 65 years, hypertension, diabetes mellitus, ischemic heart disease, underlying liver disease, chronic kidney disease (eGFR ≤ 60 ml/min/1.73 m^2^), and AKI stage.

*AKI* acute kidney injury, *HTN* hypertension, *DM* diabetes mellitus, *CKD* chronic kidney disease.

### Mortality of obstructive uropathy

The overall mortality rate during the median follow-up period was 34%, with the highest rate observed in malignancy-associated obstruction (52%), followed by other causes (15.4%) and urolithiasis (8.9%) (p < 0.001). The mortality rate was significantly higher in patients with AKI, regardless of the etiology of the obstruction (Table 6).

**Table 6:** Mortality of obstructive uropathy.

	Malignancy (n-936)		Urolithiasis (n = 481)		Others (n = 266)		p-value
	NonAKI (n = 162)	AKI (n = 774)	NonAKI (n = 134)	AKI (n = 347)	NonAKI (n = 72)	AKI (n = 194)	
Mortality	70 (43.2%)	417 (53.9%)	2 (1.5%)	41 (11.8%)	3 (4.2%)	38 (19.6%)	< 0.001

*AKI* acute kidney injury.

**Figure 2:**
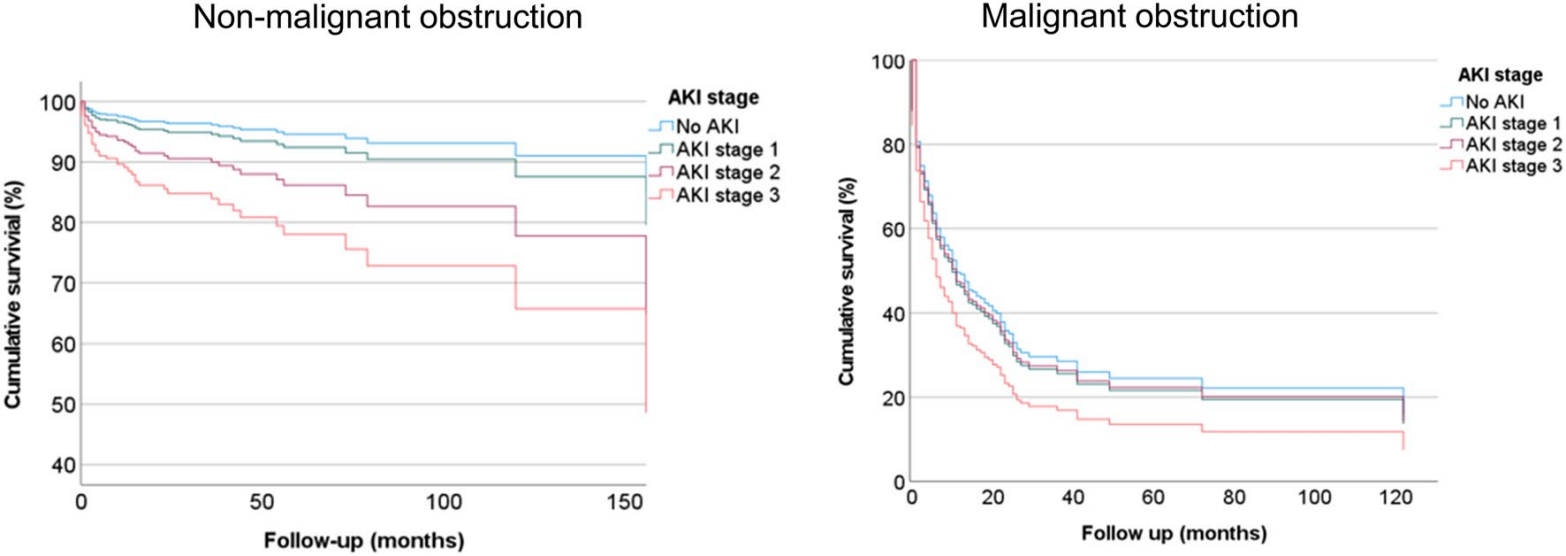
Cumulative survival of patients with obstructive uropathy according to AKI. The cumulative survival rates varied significantly with the AKI phase in non-malignancy related PCN. However, it was not different in malignancy related PCN.

We also separately analyzed malignancy *vs.* non-malignancy-associated obstructive uropathy to identify independent risk factors for mortality. Cox regression analysis showed that older age (OR, 3.49; 95% CI 1.71–7.1; p < 0.001), male sex (OR, 2.07; 95% CI 1.12–3.83; p = 0.02), lower albumin level (OR, 0.28; 95% CI 0.17–0.46; p < 0.001), and stage 3 AKI (OR, 2.28; 95% CI 1.26–4.14; p = 0.01) were found to be independent predictors of overall mortality in non-malignancy-associated obstructive uropathy (Table 7). The Kaplan–Meier survival curve also showed a stepwise decrease in the cumulative survival as the KDIGO AKI stage increased (79.6% with no AKI, 67.1% with stage 1 AKI, 65% with stage 2 AKI, and 55.8% with stage 3 AKI) (Fig. 2). In contrast, the cumulative survival did not differ significantly according to the KDIGO stage of AKI in patients with malignancy-associated obstructive uropathy. Only low albumin level (OR, 0.62; 95% CI 0.49–0.97; p = 0.03) was found to be an independent factor predicting mortality in these patients (Table 8).

**Table 7:** Cox regression analysis of factors associates with mortality (non-malignant obstruction).

	Univariate			Multivariate		
	OR	95% CI	p-value	OR	95% CI	p-value
Age	03.02	1.86–4.91	0.85	3.49	1.71–7.1	< 0.001
Sex	1.81	1.15–2.84	0.01	2.07	1.12–3.83	0.02
HTN	1.17	0.75–1.86	0.50			
DM	1.29	0.83–2.02	0.25			
CKD	1.56	1.01–2.48	0.03	1.37	0.59–3.18	0.47
Hb	2.5	1.56–4.02	< 0.001	0.96	0.82–1.11	0.57
CRP	1.00	0.92–1.01	0.13	1.00	0.99–1.00	0.07
Albumin	0.38	0.27–0.51	< 0.001	0.28	0.17–0.46	< 0.001
Hyperuricemia (serum uric acid ≥ 6.0 mg/dL)	1.09	1.09–3.23	0.02	1.02	0.56–2.14	0.79
AKI stage 3	2.97	1.94–4.57	< 0.001	2.28	1.26–4.14	0.01

Multivariate Cox regression analysis was conducted after adjusting for sex, age over 65 years, hypertension, diabetes mellitus, ischemic heart disease, underlying liver disease, chronic kidney disease (eGFR ≤ 60 ml/min/1.73 m^2^), KDIGO stage 3 AKI, Hb < 11 g/dL, CRP, albumin, and uric acid.

*HTN* hypertension, *DM* diabetes mellitus, *CKD* chronic kidney disease, *Hb* hemoglobin, *CRP* C-reactive protein.

**Table 8:** Cox regression analysis of factors associated with mortality (malignant obstruction).

	Univariate			Multivariate		
	OR	95% CI	p-value	OR	95% CI	p-value
Age	1.18	0.98–1.41	0.08	0.99	0.98–1.00	0.08
Sex	0.98	0.82–1.18	0.85			
HTN	1.17	0.98–1.41	0.09			
DM	1.01	0.82–1.24	0.95			
CKD	1.20	0.96–1.51	0.11	1.23	0.83–1.83	0.29
AKI stage 3	1.33	1.11–1.61	0.01	1.01	0.64–1.75	0.83
Albumin	0.61	0.51–0.74	< 0.001	0.62	0.49–0.97	0.03

Multivariate Cox regression analysis was conducted after adjustment for sex, age over 65 years, hypertension, diabetes mellitus, ischemic heart disease, underlying liver disease, chronic kidney disease (eGFR ≤ 60 ml/min/1.73 m^2^), KDIGO stage 3 AKI, Hb < 11 g/dL, CRP, and albumin.

*AKI* acute kidney disease, *HTN* hypertension, *DM* diabetes mellitus, *CKD* chronic kidney disease.

## Discussion

This multicenter, retrospective study demonstrated that the presence of AKI negatively affects the long-term outcomes of patients with who underwent PCN due to obstruction. The major findings of our study were as follows: (1) the most common cause of obstructive uropathy in adults was malignancy, followed by urolithiasis and other causes; (2) a substantial portion (78%) of patients presented with AKI, concomitant infection and short-term recovery after PCN insertion was achieved in 56.8% of these patients; (3) 4.4% of patients in the urolithiasis group and 6.8% in other causes group progressed to ESKD; (4) the overall mortality rate was 34%, with the highest mortality attributed to malignancy-associated obstruction (52%); and (5) stage 3 AKI was independently associated with both ESKD progression and mortality only in non-malignant causes of obstruction.

Obstruction to urine flow can occur at any site of the urinary tract. Clinical manifestations depend on the etiology, location, and duration of obstruction, as well as the presence of underlying comorbidities; thus, they can vary widely, ranging from asymptomatic cases to renal colic, urosepsis, and dialysis requiring AKI or ESKD. In the pediatric population, obstructive uropathy from congenital anomalies of the urinary tract is responsible for 30–50% of ESKD cases. However, data on the epidemiology of obstructive uropathy in the adult population are limited^2^.

This study aimed to investigate the clinical characteristics and outcomes of obstructive uropathy. PCN or pyelonephrostomy is an interventional procedure for the decompression of collecting systems and has become the primary option for temporary relief of obstruction in upper urinary tract obstruction since its first introduction in 1955^7^. We first demonstrated that the most common causes of obstructive uropathy in adults undergoing PCN were malignancy (55.6%), followed by urolithiasis (28.7%), and other causes (15.2%). In contrast with a previous study that demonstrated that uterine cervical and bladder cancer are the most common malignancy types, metastatic colorectal and gastric cancers were responsible for 46.3% of cases in our study^8^. This might be due to the exclusion of patients with urogenital cancer-associated lower tract obstruction, which was likely to be relieved with bladder decompression, or due to geographical differences in prevalent cancer types. Obstructive uropathy from direct tumor invasion or metastatic retroperitoneal lymphadenopathy is usually considered an ominous sign, and as expected, the overall mortality from malignancy-associated obstructive uropathy was higher (52%) than that from urolithiasis or other causes.

Patient characteristics varied significantly according to the different etiologies of obstruction. Patients with malignant obstruction were significantly older, male, had lower hemoglobin levels, and were more likely to be associated with AKI despite a preserved baseline renal function. In contrast, patients with urolithiasis had a higher prevalence of diabetes, hypertension, ischemic heart disease, dementia, or hemiparesis, reflecting the close association between metabolic syndrome and urolithiasis. The prevalence of urolithiasis has been shown to have doubled in the United States in the recent years. Experimental studies have shown that type 2 diabetes and obesity facilitate lithogenesis by altering the proximal tubule handling of sodium and uric acid, and by lowering the urine pH^9–11^. The association between hypertension and urolithiasis has also been demonstrated in a population-based study^12^. Our study confirmed the association of urolithiasis not only with diabetes and hypertension but also with various cardiovascular and cerebrovascular diseases. Significantly higher CRP, procalcitonin levels, and leukocyte counts in this group suggested that stone-related obstructive uropathy might be associated with superimposed infection of the urinary tract. In contrast, obstructive uropathy from other causes was characterized by a female predominance, young age, and a higher prevalence of preexisting CKD.

The present study demonstrated that 78.1% of patients initially presented with AKI, suggesting that obstructive uropathy is frequently complicated by AKI. However, given that PCN is usually indicated upon renal functional deterioration or pyonephrosis, it could simply indicate that AKI is one of the most common indication of PCN in obstructive uropathy. In general, obstruction of a single kidney does not lead to AKI if the contralateral kidney function is normal. However, because we did not analyze the dual obstruction, it was difficult to classify the exact causes of AKI as predominantly postrenal or multifactorial. Given that obstructive uropathy is frequently complicated by urinary tract infection, sepsis, or combined volume depletion, the mechanisms underlying AKI development in this setting seem to be more complex. Among the many factors with significant differences, preexisting CKD, hyponatremia (≤ 135 mEq/L), and higher uric acid levels were independently associated with AKI.

Kidney outcomes in obstructive uropathy primarily depend on the prompt relief of obstruction. Therefore, delayed diagnosis due to the absence of specific signs and symptoms or underlying comorbidities might affect recovery. An estimated GFR of ≥ 60 ml/min/1.73 m^2^, recovery to < 25% of baseline eGFR, or dialysis independence within 7 days after PCN were defined as short-term recovery. Functional recovery was achieved in 56.8% of the patients, and the underlying etiologies of obstruction did not affect the short-term recovery rates. The presence of CKD was an independent factor associated with the non-recovery of short-term renal function. This is comparable with data by *Khalaf *et al. that preoperative GFR is an independent factor affecting the functional recovery in a prospective study of 91-patients with unilateral obstructive uropathy^13^. However, the lack of information about the exact duration of obstruction, which is the single-most important factor for recovery, was one of the limitations of our study, resulting in a possibility that failed short-term recovery in 43.2% of patients is related to delayed timing of relief of obstruction. Failure to determine the regression of hydronephrosis was another limitation of this study.

Because only a few studies have determined the long-term outcome of obstructive uropathy in adults, we examined the ESKD progression and mortality. Using data from both the ESKD registry of the Korean Society of Nephrology and the hospitals, we found that 45 (2.67%) patients progressed to ESKD during the median follow-up period of 91 [range 12–219] months. The progression rate in patients with urolithiasis/other causes was 4.4% and 6.8%, respectively, while that from malignancy was only 0.6%. The very low progression rate might be due to the high premature death rate from malignancy-related complications. According to the United States Renal Data System (USRDS), urolithiasis accounts for only 0.2% of the incidents of ESRD^14^. However, El-Zoghby et al. demonstrated that the attributable risk of ESKD from symptomatic urolithiasis was 5.1% in a population-based study conducted in Olmsted County, Mn, USA, which is comparable with our data^15^. As patients with urolithiasis were more likely to be diabetic, hypertensive, or have various cardio-cerebro-vascular diseases in our study, the mechanisms underlying the progression of urolithiasis seem to be complex, involving multiple comorbidities, superimposed infection, or obstruction-related mechanisms. We found that older age, pre-existing CKD, and stage 3 AKI were independently associated with ESKD. Although AKI is known to contribute to the increased burden of progressive CKD/ESKD^16,17^, most of the data came from the study of patients with intrinsic causes of AKI, such as ischemia, sepsis, or nephrotoxicity, but not from obstructive uropathy. Therefore, this is the first study to demonstrate that superimposed AKI also plays an important role in progressive CKD in obstructive uropathy. In contrast, none of these factors, such as stage 3 AKI, affect the progression of malignant obstruction. Stage 3 AKI complicated by obstructive uropathy in our study was independently associated with overall mortality in patients with non-malignant obstruction. Older age, male sex, and hypoalbuminemia were found to predict mortality. However, similar to ESKD progression, none of these factors were associated with mortality in malignant obstruction, in which patients are likely to die from cancer-related complications.

Despite several novel findings, our study had some limitations. This was a retrospective study of patients with predominantly upper urinary tract obstruction who received PCN. Another limitation is the exclusion of common causes of obstructive uropathy, such as benign prostatic hypertrophy. In addition, the complex mechanisms that lead to AKI were not determined. However, to our knowledge, this is the first large-scale clinical study to analyze various etiologies, clinical manifestations, short- and long-term outcomes, and related risk factors in adult obstructive uropathy. With the increase in the aging population with multiple comorbidities, the incidence and outcome of adult obstructive uropathy are likely to change. A better understanding of the epidemiology and prognostic factors is needed for the development of proper management strategies for obstructive uropathy.

## Methods

### Study subjects

We retrospectively screened the medical records of 2127 patients who underwent PCN for obstructive uropathy between January 1, 2002 and December 31, 2018, in three tertiary hospitals in Korea. Four hundred forty four patients who were under age of 18 years, diagnosed with ESKD or died within 48 h of admission or obstruction due to BPH were excluded. Because lower urinary tract obstruction is frequently relieved by bladder decompression, we excluded those with obstruction due to BPH or other urethral pathologies. The study was approved by the Institutional Review Board of Korea University Anam Hospital (IRB number 2019AN0403) and conducted according to the criteria set by the Declaration of Helsinki. This study was a retrospective medical chart review, so informed consent was waived by Institutional Review Board of Korea University Anam Hospital.

### Baseline demographics and clinical characteristics

Demographic features, and clinical and laboratory data of the patients at the time of admission were reviewed. The presence of comorbidities was identified using the International Classification of Disease-9 (ICD-9) code. Estimated GFR was calculated using the Chronic Kidney Disease Epidemiology Collaboration (CKD-EPI) equation, and a prior history of eGFR < 60 ml/min/1.73 m^2^ for ≥ 3 months was considered as CKD. Baseline kidney function was defined as eGFR determined within 3 months before admission or the highest eGFR recorded during the follow-up period when baseline serum creatinine was not available. AKI was defined and staged according to the Kidney Disease Improving Global Outcomes (KDIGO)^18^.

### Outcomes

Patients were followed up until December 2019. The median follow-up period for mortality and ESKD progression was 83 months (range 0–219 months) and 91 months (range 12–219 months), respectively. Short-term recovery was defined as the recovery of kidney function to either eGFR ≥ 60 ml/min/1.73 m^2^, ≤ 25% of baseline eGFR, or attainment of dialysis independence within 7 days after PCN insertion. Patients were classified as progressing to ESKD if they needed maintenance hemodialysis, peritoneal dialysis, or kidney transplantation. ESKD data were obtained from the Korean Society of Nephrology ESKD Registry. Mortality data were obtained from hospital records and the National Database of Statistics, Korea.

### Statistical analysis

Continuous variables with standard distributions were described as the mean ± standard deviation using Student’s t-test. If the variable distributions were skewed, they were described as medians (minimum–maximum). Categorical variables were analyzed using the Chi-square test. One-way analysis of variance with Bonferroni post-hoc analysis was used to compare two or more groups. A decision tree model was used to identify the risk factors for AKI and performed logistic regression analyses to assess the risk factors for AKI or recovery from AKI. Cox regression analysis was used to explore the risk factors of mortality. Statistical analyses were performed using IBM SPSS Statistics for Windows, version 28.0. Statistical significance was set at p < 0.05.

## Supplementary Information

Supplementary Information.
