# Supplementary material for: Impact of acute kidney injury on long-term adverse outcomes in obstructive uropathy
Source: Sci Rep. 2021 Dec 8;11:23639. doi: 10.1038/s41598-021-03033-0 (PMC8654816; doi:10.1038/s41598-021-03033-0)
Supplement: Supplementary file 1 — Supplementary Information. [file 41598_2021_3033_MOESM1_ESM.pdf]

Supplementary Table S1. Type of malignancies causing ureteral obstruction with percutaneous nephrostomy

Malignancy	Frequency, n (%)	Mortality, n (%)
Colorectal Ca.	245 (26.2)	131 (53.5)
Gastric Ca.	188 (20.1)	128 (68.1)
Bladder Ca.	143 (15.3)	55 (38.5)
Cervical Ca.	97 (10.4)	46 (47.4)
Prostate Ca.	68 (7.3)	34 (50.0)
Ovarian Ca.	32 (3.4)	15 (46.9)
Ureter Ca.	30 (3.2)	10 (33.3)
Lymphoma	21 (2.2)	14 (66.7)
Pancreatic Ca.	17 (1.8)	9 (52.9)
Breast Ca.	13 (1.4)	9 (69.2)
Sarcoma	12 (1.3)	6 (50.0)
Lung Ca.	10 (1.1)	5 (50.0)
Renal cell Ca.	4 (0.4)	4 (100)
Others	56 (6.0)	30 (53.6)
Total No.	936 (100)	496 (100)

Supplementary Table S2. Logistic regression analysis of factors predicting nonrecovery of renal function by day 7 after percutaneous nephrostomy insertion

	Univariate			Multivariate		
	HR	95% CI	p-value	HR	95% CI	p-value
Age	1.00	0.99-1.003	0.271			
Sex	1.20	0.96-1.50	0.105			
HTN	1.03	0.82-1.28	0.808			
DM	1.05	0.82-1.33	0.688			
CKD	1.31	1.03-1.69	0.031	2.0	1.01-3.93	0.04
Hb	0.99	0.93-1.04	0.07			
CRP	1.03	1.00-1.05	0.02			
Glucose (per 10mg/dl)	1.04	0.43-2.55	0.92			
Albumin	1.34	1.100-1.642	0.004			

Age, sex, DM, HTN, KDIGO AKI 3 stage, chronic kidney disease (eGFR ≤ 60 ml/min/1.73 m^2), Hb, Na, K, total CO2, total Ca, Mg, total bilirubin, albumin, uric acid, CRP, and glucose.

DM, diabetes mellitus; HTN, hypertension; CKD, chronic kidney disease; Hb, hemoglobin; CRP, C-reactive protein
